# Supplementary material for: Facile Synthesis of Nitrogen-Doped Carbon Quantum Dots with Chitosan for Fluorescent Detection of Fe3+
Source: Polymers (Basel). 2019 Oct 23;11(11):1731. doi: 10.3390/polym11111731 (PMC6918340; doi:10.3390/polym11111731)
Supplement: Supplementary file 1 [file polymers-11-01731-s001.pdf]

Supplementary Materials: Facile Synthesis of Nitrogen-Doped Carbon Quantum Dots with Chitosan for Fluorescent Detection of Fe^{3+}

Li Zhao [1], Yesheng Wang [1], Xihui Zhao [1,2,*], Yujia Deng [1] and Yanzhi Xia [2]

1 School of Chemistry and Chemical Engineering, Qingdao University, Qingdao 266071, China
2 State Key Laboratory of Bio-fibers and Eco-textiles, Shandong Collaborative Innovation Center of Marine Biobased Fibers and Ecological textiles, Institute of Marine Biobased Materials, Qingdao University, Qingdao 266071, China
* Correspondence: zhaoxihui@qdu.edu.cn

Synthesis of N-CQDs

At first, 0.5 g chitosan was dissolved in 100 mL of 1% acetic acid solution, and then the chitosan solution was filtered with an aperture of 0.45 μm to remove the insoluble substance. Secondly, 20 mL of 0.5% chitosan solution was placed in an autoclave (50 mL) and heated at 180 °C for a certain time (2–24 h). After the reaction,, the reactor was cooled down to room temperature. The brownish black solution was filtered by a membrane with an aperture of 0.22 μm. Next the solution was centrifuged at 10,000 rpm for 15 min to remove all deposits and obtain a canary yellow N-CQDs aqueous solution, and then the as-prepared N-CQDs were stored in a refrigerator at 4 ◦C for future use.

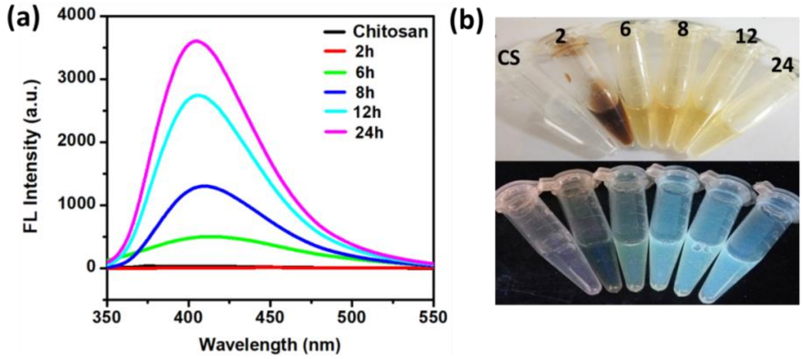

Figure S1. (**a**) Fluorescence emission spectra of N-CQDs obtained by chitosan hydrothermal carbonization with different times (at a maximum excitation wavelength of 330 nm). (**b**) From left to right are photographs of chitosan, N-CQDs with carbonization time 2, 6, 8, 12 and 24 h illuminated by natural light (Top) and UV lamp with 365 nm light (Down).

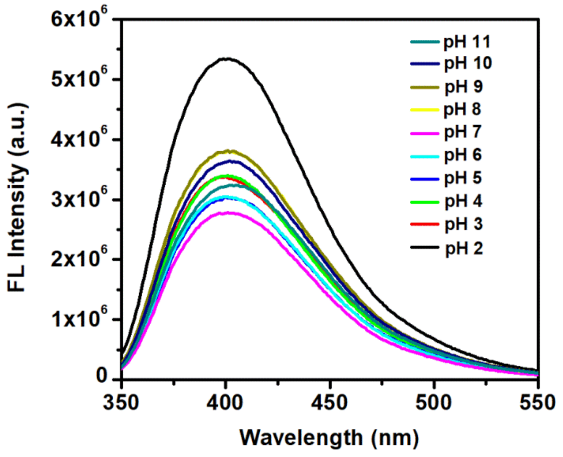

Figure S2. Effect of pH from 2 to 11 on the fluorescence intensity of N-CQDs with 500 μM of Fe^{3+} ion.

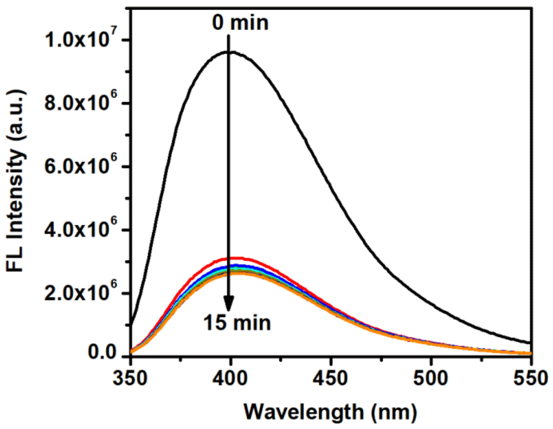

Figure S3. Effect of reaction time on the fluorescence intensity of N-CQDs with 500 μM of Fe^{3+} ion.

Table S1. Determination of Fe^{3+} in tap water and waste water samples.

Samples		Concerntration of Fe^{3+} (μM)		Recovery (%)	RSD (n = 3, %)
		Spiked	Measured		
Tap water	1	0	0.5	-	1.20
	2	2.0	2.42	96.8	1.14
	3	5.0	5.53	100.5	3.75
	4	15.0	15.35	99.0	2.40
Lake water	1	0	0.57	-	1.76
	2	2.0	2.62	101.9	2.11
	3	5.0	5.54	99.5	1.73
	4	15.0	15.45	99.2	1.68

Recovery = $[(C_2 - C_1)/C_0] \times 100\%$

Where C_0 is the concentration of Fe^{3+} added into the real samples, C_1 and C_2 is the concentration of Fe^{3+} in real samples before and after adding the standard Fe^{3+}.
